# Childhood Polyarthritis As Early Manifestation of Autoimmune Polyendocrinopathy with Candidiasis and Ectodermal Dystrophy Syndrome

**DOI:** 10.3389/fimmu.2017.00377

**Published:** 2017-04-18

**Authors:** Maria J. Gutierrez, Julieta Gilson, Jamie Zacharias, Faoud Ishmael, C. April Bingham

**Affiliations:** ^1^Division of Pediatric Allergy Immunology, Johns Hopkins University School of Medicine, Baltimore, MD, USA; ^2^Naples Community Hospital, Naples, FL, USA; ^3^Section of Allergy, Asthma and Immunology, Pennsylvania State University College of Medicine, Hershey, PA, USA; ^4^Division of Pediatric Rheumatology, Penn State Children’s Hospital, Hershey, PA, USA

**Keywords:** autoimmune polyendocrinopathy with candidiasis and ectodermal dystrophy, autoimmune polyendocrinopathy syndrome I, arthtritis, hypoparathyroidism, autoimmune regulator, chronic mucocutaneous candidiasis

## Abstract

Autoimmune polyendocrinopathy with candidiasis and ectodermal dystrophy (APECED) is a rare disorder of immune dysregulation caused by mutations in the autoimmune regulator (*AIRE*) gene. Individuals affected with APECED develop a clinical syndrome characterized by ectodermal abnormalities, autoantibody production, and organ-specific autoimmune manifestations. Inflammatory arthritis is usually not described as a part of the syndrome, and only sporadic cases are reported. We describe the case of a preschool-age girl who presented with hypoparathyroidism, hepatitis, interstitial pneumonitis, and chronic polyarthritis at 4 years of age and was found to have two compound heterozygous disease-associated mutations in the *AIRE* gene. We also conducted a literature review of the main characteristics of inflammatory arthritis in APECED patients. Our case and review demonstrate that (1) inflammatory arthritis, although rare, can be an early manifestation of APECED; (2) the diagnosis of APECED should be considered if mucocutaneous candidiasis, multiple organ-specific autoimmune manifestations, polyendocrinopathy, especially hypoparathyroidism or adrenal failure, or ectodermal dystrophy accompany joint symptoms; and (3) genotyping interpretation should take into account that mutations are found in the 14 exons of the gene, compound heterozygosity is common, and in some cases, only one or no mutated alleles are found.

## Background

Autoimmune polyendocrinopathy with candidiasis and ectodermal dystrophy (APECED) is a rare disorder of immune dysregulation caused by mutations in the autoimmune regulator (*AIRE*) gene (1). *AIRE* is a master transcriptional regulator with a critical role in generating central immune tolerance within the thymus (2, 3). The *AIRE* gene maps to chromosome 21.q.22.3 and is composed of 14 exons that encode a 545-amino-acid protein. Approximately 104 mutations in all 14 exons have been characterized in association with APECED (4). Although disease manifestations show geographical variation (5), no definitive genotype–phenotype correlations have been established (6).

In healthy individuals, *AIRE* promotes the expression of tissue-specific antigens (TSAs) in medullary thymic epithelial cells (mTECs) (7, 8). T-cells that recognize these TSAs with high affinity are deemed autoreactive and suffer negative selection (7). *AIRE* also influences the positive selection of certain CD4+FOXP3+ and CD8+CD28+ regulatory T-cells, modulates chemokine expression involved in thymocyte egress, and may promote apoptosis of mTECs, thereby further promoting autoantigen presentation (9). More recently, concomitant expression of *AIRE* and TSAs has also been detected in thymic B-cells, suggesting an additional role of *AIRE* in B-cell-mediated central T-cell tolerance (10, 11). Outside the thymus, *AIRE* is expressed by blood monocytes and dendritic cells, likely also playing a role in the induction of peripheral self-tolerance (12). As a result of mutation in the *AIRE* gene, APECED-affected individuals feature a clinical syndrome characterized by autoantibody production and endocrine and non-endocrine autoimmune manifestations (13–15).

The classic symptom triad consists of chronic mucocutaneous candidiasis (CMC), hypoparathyroidism, and autoimmune adrenal insufficiency (13–15). Traditionally, the diagnosis of APECED has required that at least two of these major components are present or, alternatively, one component if a sibling is affected (16). Nonetheless, up to 80% of patients develop non-triad manifestations before diagnostic criteria are met, and an adjunct diagnostic triad of enamel hypoplasia, gastrointestinal dysfunction, and urticarial eruption has been recently proposed to identify those early cases (5). Additional endocrine gland involvement is common, which leads to hypogonadism, hypothyroidism, growth hormone deficiency, and type I diabetes. Non-endocrine manifestations include pernicious anemia, inflammatory eye disease, asplenia, tubulointerstitial nephritis, sicca syndrome, alopecia, and vitiligo (15, 17). Non-inflammatory ectodermal abnormalities such as nail dystrophy and calcification of the tympanic membrane are also seen (17). Importantly, serious and life-threatening manifestations such as pneumonitis and autoimmune hepatitis (both seen in up to 43% of patients) may appear at any time in the course of the disease (5).

Autoimmune polyendocrinopathy with candidiasis and ectodermal dystrophy is traditionally regarded as a disorder of immune dysregulation with predominantly organ-specific autoimmune manifestations (9, 15). Arthritis is not described as a part of the syndrome in most of the large APECED cohorts (5, 14, 18–23), and only sporadic cases are reported (24–31). Here, we describe the case of a preschool-age girl who presented with hypoparathyroidism, hepatitis, interstitial pneumonitis, and chronic polyarthritis at 4 years of age and was found to have two heterozygous disease-associated mutations in the *AIRE* gene. We also conducted a comprehensive review of reported cases of inflammatory arthritis in APECED patients.

## Case Presentation

At age 4.5 years, a US-born girl was referred to our *Pediatric Rheumatology* clinic with an approximate 6-month history of a decreased range of motion of the right wrist. She was born after an uncomplicated pregnancy to non-consanguineous parents of Eastern European descent and had an uneventful prenatal course. Medical history was significant for one previous episode of pneumonia treated with oral antibiotics at the age of 1 year. She had had a few episodes of self-resolved herpes labialis and recurrent episodes of acute otitis media requiring tympanostomy tube placement at the age of 2 years. She also had a history of mild eczema and poor dentition that required multiple dental extractions. She did report intermittent chronic abdominal pain, bloating, and diarrhea. She had been diagnosed with hypoparathyroidism at the age of 3 years after an incidental finding of hypocalcemia and was receiving calcium and vitamin D supplementation. More recently, she had been admitted to our children’s hospital with an acute episode of rhinovirus-associated pneumonitis. Otherwise, she had no history of additional opportunistic, atypical, or invasive infections. Remarkably, she had no history of candidal infections. She had received all age-appropriate immunizations uneventfully. The patient lived at home with her family, and there were no additional relatives with a history of immunodeficiency or autoimmunity.

**Figure 1:**
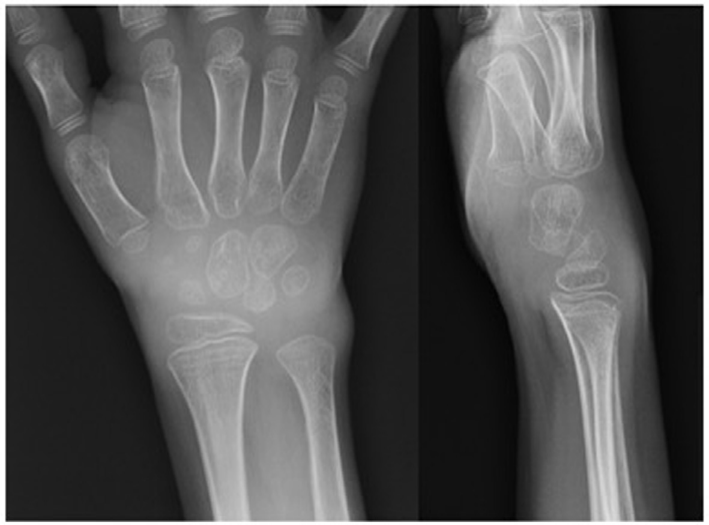
**Right wrist radiograph**. Radiographic changes consistent with damage secondary to inflammatory arthritis were observed. The patient had approximately a 6-month history of a decreased range of motion of right wrist. Widening of the scapholunate interval is noted on the AP view. Volar displacement of the capitate from the axis of the radius and lunate and volar rotation of the lunate are seen on the lateral view. Periarticular osteopenia and soft tissue swelling are seen on both views.

On examination, she was a well-developed girl. She had mild skin pallor and thin hair. Her abdomen was markedly distended with a visible venous pattern. Musculoskeletal examination demonstrated a joint effusion with a decreased range of motion of the right wrist. Joint effusions were also noted in the left wrist, left ankle, and the second and third metacarpophalangeal and proximal interphalangeal joints of the left hand. Radiographic images of the right wrist demonstrated volar displacement of the capitate and lunate bones associated with soft tissue swelling and periarticular osteopenia, changes consistent with damage secondary to inflammatory arthritis (Figure 1).

**Figure 2:**
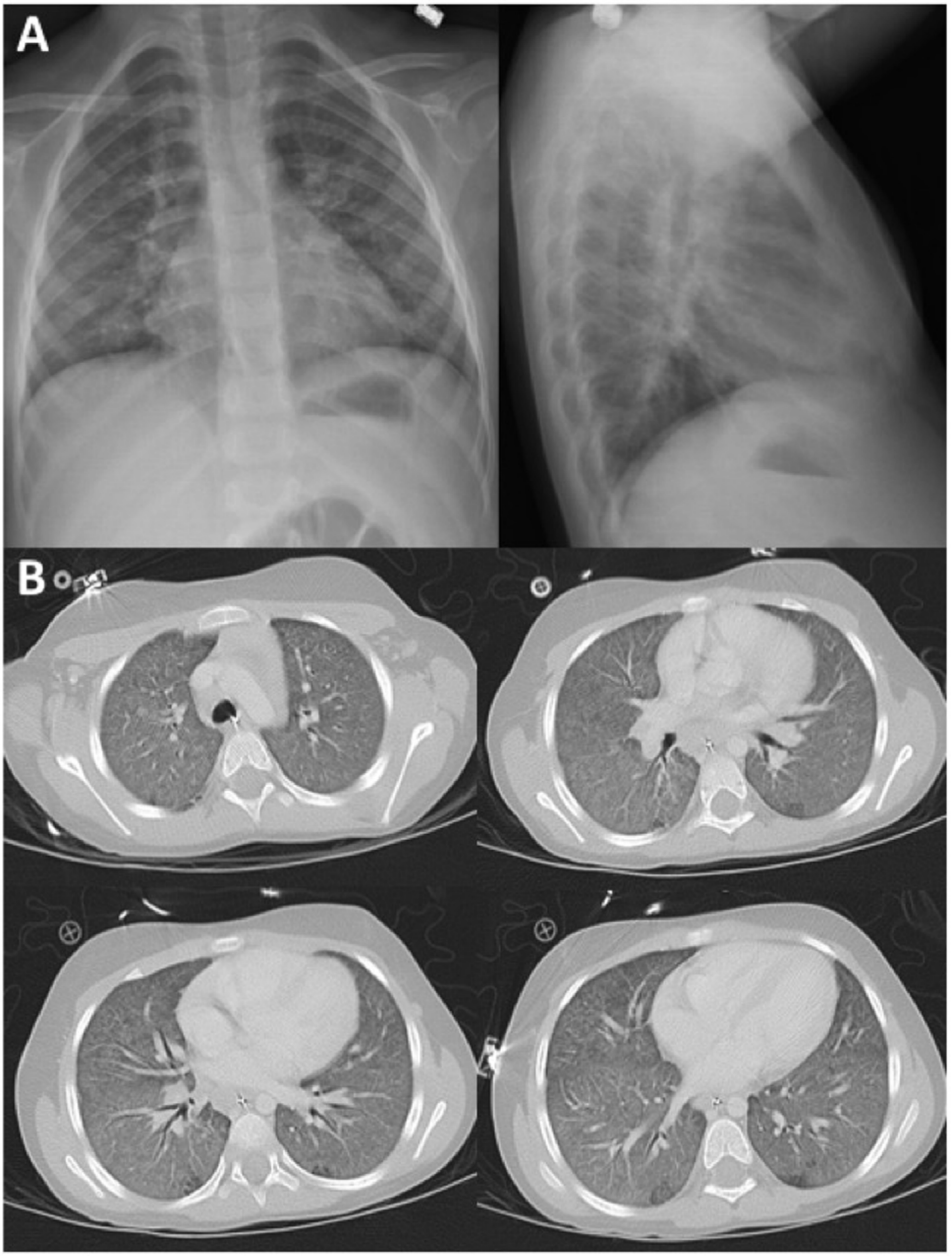
**(A)** Chest X-ray images during initial pneumonitis episode demonstrated interstitial infiltrates with ground-glass opacities of both lungs. **(B)** Chest CT scan demonstrating diffuse ground-glass opacities throughout both lungs without focal consolidation.

During a recent hospital admission, the patient had been diagnosed with pneumonitis and required oxygen supplementation for several days. Her chest X-ray demonstrated interstitial infiltrates with ground-glass opacities of the lung parenchyma (Figure 2A) confirmed by a chest computed tomography (CT) scan (Figure 2B). She had a nasal aspirate polymerase chain reaction positive for rhinovirus, and thus, a viral cause was assumed. However, because of the atypical presentation and additional abnormalities, the concern for a possible immune disorder arose.

The patient’s immunological workup (Table S1 in Supplementary Material) demonstrated normal numbers and proportions of T-, B- and natural killer cells. Lymphocyte proliferation to phytohemagluttinin and pokeweed mitogens yielded normal values. Total immunoglobulin G (IgG) and erythrocyte sedimentation rate levels were elevated. The patient had had persistent polyclonal hypergammaglobulinemia (IgG > 3,000 mg/dl) in the absence of obvious infections over several months. Immunoglobulins A, M, and E (IgA, IgM, and IgE) were normal. Pneumococcal antibody titers were non-protective at baseline, but the patient had an adequate response after the pneumococcal polysaccharide vaccine (PPSV23) was administered. She had mild microcytic anemia, and liver enzymes were markedly elevated (Table S1 in Supplementary Material). A high titer antinuclear antibody titer (>1:640) was demonstrated. Anti-Sjogren syndrome type A and B, anti-Smith, anti-ribonucleoprotein, anti-double-stranded deoxyribonucleic acid, and anti-scleroderma-70 antibodies were negative. Rheumatoid factor, anti-neutrophil cytoplasmic antibody, and angiotensin-converting enzyme levels were normal/negative. Anti-interferon-omega antibodies were not tested. Fluorescence *in situ* hybridization analysis for chromosome 22q.11 deletions was normal.

Upon review of other previous laboratory tests, additional abnormalities were noticed. In the last year, she had had chronically elevated liver enzymes. Workup for hepatitis A, B, C, and Epstein–Barr viruses had yielded no signs of active infection. Anti-gliadin and anti-tissue transglutaminase IgA as well as anti-kidney-liver microsomal and anti-smooth muscle antibodies had been negative. Liver ultrasound was normal other than sludge in gallbladder. A previous abdominal CT scan had revealed marked colonic loop distension and gallbladder sludge. The cause of her intestinal and liver abnormalities remained undiagnosed, and a liver biopsy was scheduled.

A liver biopsy demonstrated brisk lymphohistiocytic infiltrate in the portal tracts with moderate interface hepatitis. Occasional eosinophils and neutrophils were present, but plasma cells were scarce or absent. The bile duct epithelium was intact. No cholestasis or steatosis was seen. Trichrome and reticulin stains demonstrated mild fibrous expansion of some portal tracts but no nodule formation. The lobule demonstrated areas of confluent perivenular necrosis. Periodic acid–Schiff–diastase stain did not show intracytoplasmic inclusions within hepatocytes. There was no stainable iron. Despite a lack of abundance of plasma cells, this was felt clinically to be autoimmune hepatitis.

She received therapy for autoimmune hepatitis with oral prednisone with normalization of liver enzymes after approximately 4 weeks. In addition, oral methotrexate (MTX) was started as treatment for her inflammatory arthritis and autoimmune hepatitis, as this drug was felt to be the best steroid-sparing immunosuppressive medication to treat both problems. Of note, her liver enzymes remained normal while on MTX therapy. The patient had significant clinical improvement in her joint examination several months after starting MTX. There was no sign of active arthritis after 10 months of therapy. A follow-up chest CT scan 1 year after pneumonitis episode showed subtle and somewhat diffuse, centrilobular and perilymphatic nodules with scattered areas of ground-glass opacities in a non-specific pattern. Ground-glass opacities seen previously were improved. In regards to additional endocrine abnormalities, she was found to have markedly elevated anti-thyroid peroxidase (anti-TPO) and anti-thyroglobulin (anti-TG) autoantibody titers (anti-TPO antibody = 794 IU/ml with normal level <5.6 IU/ml; anti-TG antibody >1,000 IU/ml with normal level <4.1 IU/ml). At 5 years of age, she developed elevated thyroid-stimulating hormone and was started on levothyroxine. In addition, she had positive intrinsic factor antibody and developed vitamin B12 deficiency. Anti-adrenal antibody was negative, and morning cortisol levels have remained normal. Hemoglobin A1C levels have remained normal.

## Genetic Analysis

**Figure 3:**
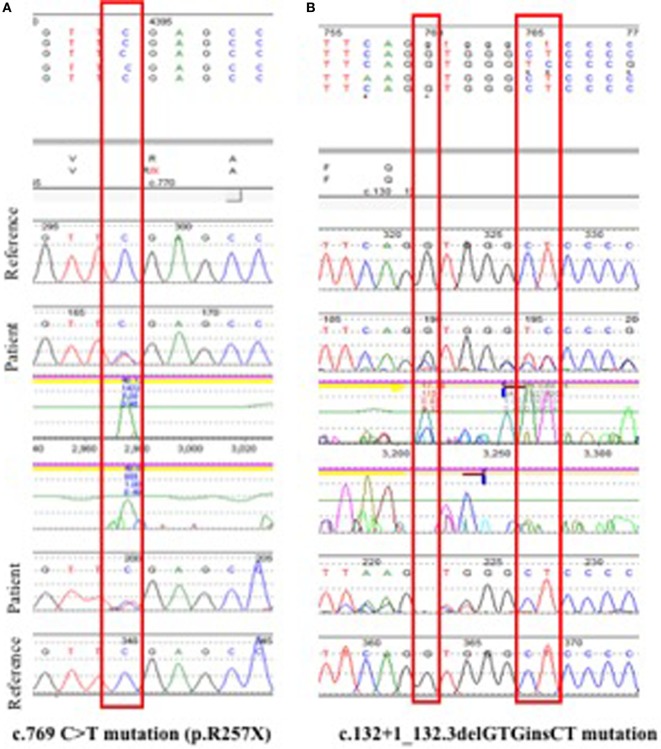
**Genotyping**. **(A)** Initial sequence analysis of exons 2, 3, 6, 7, 8, and 10 identified a non-sense heterozygous c.769C>T (R257X) mutation in exon 6 of the *AIRE* gene. This mutation is known to be disease causing and is the most common mutation found in European autoimmune polyendocrinopathy with candidiasis and ectodermal dystrophy (APECED) patients. **(B)** Subsequent sequencing of the remaining exons yielded a c.132 + 1_132 + 3delGTGinsCT splice site mutation in *AIRE*. The confirmation of *AIRE* compound heterozygosity in this patient was consistent with the clinical diagnosis of APECED.

The association of hypoparathyroidism, hypergammaglobulinemia, and immune-mediated multiorgan involvement in this patient triggered the concern for possible APECED syndrome. Initial sequence analysis of exons 2, 3, 6, 7, 8, and 10 of the *AIRE* gene in peripheral blood revealed a heterozygous c.769C>T (R257X) mutation in exon 6 (Figure 3A). This mutation affects the protein’s SAND domain and is the most commonly found mutation worldwide (6). If biallelic, the R257X mutation is predicted to cause loss of normal protein function either through protein truncation or non-sense-mediated mRNA decay (32). However, in this case, a heterozygous mutation was not confirmatory. Therefore, because of the highly suggestive clinical presentation, the next step was to genotype the *AIRE* gene exons 1,4,5,9, and 11–14, which were not sequenced initially. Indeed, a second c.132 + 1_132 + 3delGTGinsCT splice site mutation was found (Figure 3B). This mutation affects the CARD domain and is believed to destroy the canonical splice donor site in intron 1, causing abnormal gene splicing (22, 33). These findings were consistent with the diagnosis of APECED syndrome secondary to compound heterozygosity in the *AIRE* gene. The proband’s three siblings underwent genetic testing, and two of the three (siblings ages 1 and 3 years of age) were found to be compound heterozygotes with the same mutations in *AIRE* gene, but neither child had any symptoms of APECED to date. A 7-year-old sibling was found to have the R257X mutation, consistent with being a carrier of APECED. Genetic testing of the parents could not be obtained.

## Literature Review

A comprehensive literature review of reported cases of inflammatory arthritis in patients with APECED was performed using the PubMed, Embase, BIOSIS, Online Mendelian Inheritance in Man Database, and AIREbase databases from their inception through July 2016. Eligible studies included case reports, case series, case–control, cohort studies, and clinical trials that included clinical descriptions of patients with APECED. Our search yielded nine reports of arthritis in patients with APECED (Table 1) (13, 24–31). There were seven cases of childhood-onset arthritis (22, 25–31) and two cases of adult-onset rheumatoid arthritis (RA) reported (13, 24). Most pediatric patients developed arthritis in early childhood (under 5 years of age). Strikingly, in four patients, the joint symptoms preceded the development of additional autoimmune complications by several years (25, 27, 28, 31). Specific juvenile idiopathic arthtritis (JIA) subtypes were described in three reports (one patient had systemic-onset JIA and two had oligoarticular disease) (27, 28, 30). The JIA subtype could not be determined in the remaining cases. Genotyping was available in five cases (25, 27, 28, 30, 31). There were three compound heterozygotes (27, 28, 31) and two homozygote individuals. The treatment used for arthritis was reported in only two cases (25, 26). In one patient, arthritis resolved with the non-steroidal anti-inflammatory tolmetin after 1 year (26). A second patient was treated with MTX along with prednisone (25).

**Table 1:** **Summary of reported cases of APECED-associated arthritis**.

Reference	Gender	Age of arthritis onset (years)	Type of arthritis	*AIRE* mutation	Associated symptoms	Arthtritis treatment
Bruni et al. (26)	M	10	N/A	N/A	HP, AF, DM type I, PI, GID	NSAID (tolmetin)
Pun et al. (29)	F	3	N/A	N/A	CMC, A, HG, HP, GHD, type I DM, TOF, dental caries, cholesteatoma, seizures, AF, PA, nephrocalcinosis, GID, cataracts	N/A
von Schnurbein et al. (25)	F	1^a^	N/A	c.462G>A	CMC, HP, HT, AF, AH, V, GID	MTX, prednisone
Magitta et al. (31)	M	4^a^	N/A	c.274C>T and c.967-979*del*13	AF, HG, A	N/A
Posovszky et al. (30)	F	2	Monoarthritis	c.463G>A	AH, CMC, PA	N/A
Podkrajsek et al. (28)	F	2^a^	Systemic-onset JIA	c.892G>A and c.769C>T	HP, AF, HG, PA, chronic otitis, asthma	N/A
Meloni et al. (27)	F	2^a^	Pauciarticular JIA	c.232T>A and c.64_69del (p.V22_D23del)	HP, anti-LKM, and anti-thyroid peroxidase antibodies	N/A
Conte (24)^b^	F	42	RA	N/A	Hypocalcemia, CMC, anti-adrenal and anti-gastric parietal cell antibodies, prolonged QT, cataracts	N/A
Ahonen et. al (13)	N/A	Adult onset	RA	N/A	N/A	N/A

*^a^In these four reports, inflammatory arthritis was the first autoimmune manifestation of APECED, preceding mucocutaneous and endocrine symptoms by years*.

*^b^This case is reported under the diagnosis of familial chronic idiopathic hypoparathyroidism*.

*A, alopecia; AF, adrenal failure; AH, autoimmune hepatitis; APECED, autoimmune polyendocrinopathy with candidiasis and ectodermal dystrophy; CMC, chronic mucocutaneous candidiasis; DM, diabetes mellitus; GID, gastrointestinal dysfunction; HG, hypogonadism; HP, hypoparathyroidism; HT, hypothyroidism; JIA, juvenile idiopathic arhritis; LKM, liver kidney microsomal; MTX, methotrexate; PA, pernicious anemia; PI, pancreatic insufficiency; RA, rheumatoid arthritis; TOF, tetralogy of fallot; V, vitiligo; N/A, not available*.

## Discussion

Inflammatory arthritis is only rarely described in association with APECED syndrome and is typically absent from clinical manifestations reported in large APECED cohorts (5, 14, 18–23). Here, we present the case of a 4-year-old girl, found to have two compound heterozygous disease-associated mutations in the *AIRE* gene (c.769C>T and c.132 + 1_132 + 3delGTGinsCT), featuring polyarticular arthritis as an early manifestation of APECED. Our literature review yielded nine additional cases of APECED-associated arthritis (Table 1) (13, 24–31). Most cases describe pediatric patients with arthritis onset between 1 and 10 years of age (25–31). Arthritis subtypes are often not reported, but include systemic-onset JIA (28) and oligoarticular disease (27, 30). In contrast, our patient had polyarticular disease. Importantly, in four children, arthritis occurred before other symptoms (25, 27, 28, 31). Collectively, this evidence supports the notion that although rare, inflammatory arthritis can be a manifestation of APECED, the arthritis phenotype is variable, and it may even be the first symptom of the syndrome in children. Children with persistent joint complaints should be referred to a rheumatologist for a thorough joint examination to diagnose inflammatory arthritis. X-rays may show joint damage as our patient had; however, children with inflammatory arthritis often have normal x-rays early in disease, so radiographs are alone not adequate for evaluation.

Additional disease manifestations in this case included hypoparathyroidism, dental abnormalities, chronic diarrhea, hypergammaglobulinemia, autoimmune hepatitis, chronic pneumonitis, hypothyroidism, and pernicious anemia. Remarkably, this patient failed to meet the traditional clinical definition of APECED as she lacked a history of CMC and adrenal insufficiency, two of the classical triad components (15). This clinical presentation reinforces the idea that non-classical manifestations can occur before classic symptoms develop (5, 15). Our patient’s abdominal pain and bloating and diarrhea suggest an enteropathy, and this manifestation of APECED can often be missed unless further investigations are conducted. Importantly, this case also highlights that life-threatening complications such as hepatitis and pneumonitis may occur early in the course of the disease, thus requiring a high index of suspicion to screen and promptly diagnose and treat these complications.

Anti-interferon or anti-Th17 cytokine antibodies were not tested in our patient or her siblings. However, these tests carry high sensitivity and specificity for APECED and are available in clinical practice (34–36). Moreover, these tests can be used as an initial screening test if APECED is suspected. These autoantibodies may be present before other laboratory or clinical manifestations of APECED (37).

In our case, selective sequencing of exons 2, 3, 6, 7, 8, and 10 revealed only one of the two deleterious mutations present in this patient, and a second genetic test to sequence the remaining exons was required to confirm the diagnosis. This experience illustrates that, although APECED is a rare disorder, the diagnosis should be fully explored if multiple organ-specific autoimmune manifestations, polyendocrinopathy, especially hypoparathyroidism or adrenal failure, or ectodermal dystrophy accompany joint symptoms. A careful interpretation of genotyping results should take into account that *AIRE* mutations have been described affecting the 14 exons of the gene (4, 6), and compound heterozygosity is frequently reported (6). In addition, large defects, usually not detected by regular single gene or massively parallel sequencing methods, may require alternative approaches such as comparative genomic hybridization for their diagnosis (5). APECED typically is characterized by autosomal recessive inheritance, but autosomal dominant inheritance has been reported (38). Importantly, up to 15% of APECED patients may have only one heterozygous or no *AIRE* mutations (5).

The fact that inflammatory arthritis is only a rare occurrence in a disease characterized by widespread autoimmunity is puzzling. On the one hand, most of the *AIRE*-induced self-antigens in mTECs are tissue specific and thus associated with organ-specific rather than systemic autoimmunity (7, 8). In addition, the expression of self-antigens from synovial tissues does not seem to be particularly dependent on thymic *AIRE* (8). On the other hand, genome-wide association studies have linked *AIRE* polymorphisms to the development of RA (39–41), and the induction of extrathymic *AIRE* has been detected in the inflamed synovium of patients with RA (42), suggesting a role of *AIRE* in synovial inflammation. Whether peripheral events, for instance, the terminal differentiation of self-reactive T-cells into arthritogenic subtypes (43) or the production of autoantibodies with ameliorating potential (44), may play a role in the development of joint symptoms in APECED is unknown. In addition, there may be *AIRE*-independent genetic variants that enhance the risk of developing arthritis in APECED patients. The role of environmental triggers and individual susceptibility in the development of arthtritis in APECED patients remain intriguing topics for further research.

The treatment of APECED-associated arthritis warrants thoughtful consideration. In our case, the patient responded favorably to MTX. However, if used, its potential for causing liver toxicity and the small but existent risk of inducing interstitial lung disease need to be weighted (45). Other therapeutic options in this case included azathioprine, a first-line medication for the therapy of autoimmune hepatitis and also a disease-modifying antirheumatic drug (46, 47). However, in this case, there was a concern that this drug might not treat the inflammatory arthritis as effectively as MTX (48). In the setting of pneumonitis, rituximab could be used since, in addition to treating inflammatory arthritis, it has been reported to successfully treat lung manifestations of the disease (49). Nonetheless, rituximab may fail to treat other associated complications (50). In summary, selecting the optimal treatment for APECED-associated arthritis represents a challenge. Special attention should be given to individual circumstances, existing comorbidities, and the safety profile of each medication. Unfortunately, APECED can be a devastating condition that is difficult to treat and, in some cases, may not respond to multiple immunosuppressive therapies (50).

In summary, APECED is a phenotypically heterogeneous disease that can rarely feature inflammatory arthritis as an early manifestation and pose particular diagnostic and therapeutic challenges to rheumatologists. Delineating the link between APECED and inflammatory arthritis might provide novel insights into the role of *AIRE* in the development of arthritis and related systemic autoimmune disorders.

## Ethics Statement

As an individual case report this study is exempt from ethics committee review. Consent was obtained from the patient’s legal guardian (mother) for the publication of this case.

## Author Contributions

MG, JG, and JZ: acquisition and interpretation of data and manuscript drafting. FI: acquisition and interpretation of data and manuscript review for critically important content. CB: acquisition and interpretation of data, manuscript drafting, and review for critically important content.

## Conflict of Interest Statement

The authors declare that the research was conducted in the absence of any commercial or financial relationships that could be construed as a potential conflict of interest.

## Abbreviations

APECED, autoimmune polyendocrinopathy with candidiasis and ectodermal dystrophy; *AIRE*, autoimmune regulator; APS-I, autoimmune polyendocrinopathy syndrome I; TSAs, tissue-specific antigens; mTECs, medullar thymic epithelial cells; CMC, chronic mucocutaneous candidiasis; PCR, polymerase chain reaction; PHA, phytohemagluttinin; PWM, pokeweed mitogen; ESR, erythrocyte sedimentation rate; IgG, immunoglobulin G; IgA, immunoglobulin A; IgE, immunoglobulin E; IgM, immunoglobulin M; anti-SSA/SSB, anti-Sjogren syndrome type A and B antibodies; anti-Sm, anti-Smith antibodies; anti-RNP, anti-ribonucleoprotein antibodies; anti-dsDNA, anti-double-stranded deoxyribonucleic acid antibodies; anti-Scl70, anti-scleroderma 70 antibodies; RF, rheumatoid factor; ANCA, anti-neutrophil cytoplasmic antibody; ACE, angiotensin-converting enzyme; AGA, anti-gliadin antibodies; anti-tTG IgA, antitissue transglutaminase IgA antibodies; anti-LKM, anti-liver, kidney, muscle antibodies; ASMA, anti-smooth muscle antibodies; anti-IFN-ω, anti-interferon-omega antibodies; anti-TPO, anti-thyroid peroxidase; anti-TG, anti-thyroglobulin antibodies; TSH, thyroid-stimulating hormone; SAND domain, Sp100, AIRE-1, NucP41/75, DEAF-1 domain of the AIRE gene; OMIM, Online Mendelian Inheritance in Man Database; JIA, juvenile idiopathic arthritis; CGH, comparative genomic hybridization; GWAS, genome-wide association study; MTX, methotrexate; AZA, azathioprine.
