# Supplementary material for: Childhood Polyarthritis As Early Manifestation of Autoimmune Polyendocrinopathy with Candidiasis and Ectodermal Dystrophy Syndrome
Source: Front Immunol. 2017 Apr 18;8:377. doi: 10.3389/fimmu.2017.00377 (PMC5394715; doi:10.3389/fimmu.2017.00377)
Supplement: Supplementary file 1 [file table_1.docx]

**Table S1. Summary of Laboratory Findings**

Patient had mild chronic anemia with significantly increased IgG levels in the absence of active infection. Erythrocyte Sedimentation Rate (ESR) and total protein levels were also increased and liver enzymes had doubled in the previous month. The patient had normal IgA and IgM levels and absolute lymphocyte count, as well as lymphocyte subpopulations. Lymphocyte proliferation to phytohemagglutinin (PHA) and Pokeweed mitogen (PWM) yielded normal values. Anti-nuclear antibody (ANA) titer was elevated at high titers (>1:640). Anti-SSA/SSB, anti-Smith, anti-RNP, anti-dsDNA, anti-Scl70 antibodies, AGA, tTG-IgA, anti- LKM, ASMA and ANCA antibodies as well as Rheumatoid Factor (RF) were negative. In contrast, anti-TPO and anti-TG were markedly increased.

| **Test** | **Patient’s results** | **Age-matched control range** |
| --- | --- | --- |
| White Cell Count | 7.53 K/uL | 4-10.4 K/uL |
| Hemoglobin | 11.0 g/dL | 11.5-13.5 g/dL |
| Platelets | 198 K/uL | 172-440 K/uL |
| Neutrophil count | 4.3 K/uL | 1.5-8.5 K/uL |
| Lymphocyte count | 2.52 K/uL | 1.5-8 K/uL |
| CD3+ T cells | 69% | 56-75% |
| CD3+CD4+ T cells | 39% | 28-47% |
| CD3+ CD8+ T cells | 29% | 16-30% |
| CD19+ B cells | 23% | 14-33% |
| CD16+CD56+ NK cells | 7% | 4-17% |
| IgG | 3635 mg/dL | 460-1240 mg/dL |
| IgA | 102 mg/dL | 25-160 mg/dL |
| IgM | 152 mg/dL | 45-200 mg/dL |
| IgE | 9.2 mg/dL | <115 mg/dL |
| ESR | 43 mm/hr | 0-10 mm/hr |
| AST | 354 unit/L | 15-46 unit/L |
| ALT | 561 unit/L | 13-69 unit/L |
| Albumin | 4.5 g/dL | 3.5-5 g/dL |
| Total Protein | 9.4 g/dL | 6.3-8.2 g/dL |
| ANA (IFA) | >1:640 | <1:40 |
| Anti-TPO | 794 I.U/ml | <5.6 I.U/ml |
| Anti-TG | >1000 I.U/ml | <4.1 I.U/ml |
| Anti-SSA/SSB, anti-Smith, anti-RNP, anti-dsDNA, anti-Scl70 antibodies | Negative | Negative |
| ANCA | Negative | Negative |
| Rheumatoid Factor | Negative | Negative |
| AGA, tTG-IgA, anti- LKM, ASMA antibodies | Negative | Negative |

Abbreviations: IgG= Immunoglobulin G, IgA=Immunoglobulin A, IgM= Immunoglobulin M, IgE=Immunoglobulin E. AST=Apartate aminotransferase, ALT= Alanine transaminase. Anti-SSA/SSB= Anti-Sjogren Syndrome type A and B antibodies, Anti-Sm= Anti-Smith antibodies, Anti-RNP= Anti-Ribonucleoprotein antibodies, Anti-dsDNA= Anti-Double Stranded Dexoyribonucleic Acid antibodies, Anti-Scl70=Anti-Scleroderma 70 antibodies, ANCA= Anti-Neutrophil Cytoplasmic Antibody. AGA= Anti-Gliadin antibodies. Anti-tTG IgA= Anti-Tissue Transglutaminase IgA antibodies, Anti-LKM= Anti-Liver, Kidney, Muscle antibodies, ASMA= Anti-Smooth Muscle antibodies, Anti-TPO= Anti-Thyroidperoxidase antibodies, Anti-TG= Anti-Thyroglobulin antibodies.
